# Prevalence and associated factors of clinical manifestations of vitamin a deficiency among preschool children in asgede-tsimbla rural district, north Ethiopia, a community based cross sectional study

**DOI:** 10.1186/s13690-016-0122-3

**Published:** 2016-03-14

**Authors:** Tesfalem Abrha, Yonas Girma, Kebede Haile, Mezgebe Hailu, Mengistu Hailemariam

**Affiliations:** Department of Medicine, Aksum University, Aksum, Ethiopia; Department of Nursing, School of Nursing and Midwifery, Tehran University of Medical Sciences, International Campus (TUMS-IC), Tehran, Iran; Center of International Reproductive Health Training (CIRHT), Bahir Dar University, Bahir Dar, Ethiopia; Department of Public Health, Aksum University, Aksum, Ethiopia; Center of International Reproductive Health Training (CIRHT) Program Manager, Addis Ababa, Ethiopia

**Keywords:** Vitamin a deficiency, Clinical vitamin a status, Night blindness, Bitot‘s spot

## Abstract

**Background:**

Vitamin A Deficiency is a common form of micronutrient deficiency, globally affecting 33.3 % of preschool-age children. An estimated of 44.4 % of preschool children in Africa were at risk for vitamin A deficiency. In Ethiopia, vitamin A deficiency leads to 80,000 deaths a year and affects 61 % of preschool children. The aim of this study was to investigate the prevalence and associated factors with the night blindness, Bitot’s spot and vitamin A intake among preschool children in rural area, Asgede-Tsimbla district, North Ethiopia.

**Methods:**

Community based cross sectional study was conducted from January 27 to March 7, 2014. A total 1230 preschool children were selected by systematic random sampling from 8 randomly selected kebelles (smallest administrative unit). Structured and pretested questionnaires adapted from relevant studies and WHO/FAO was for data collection. In addition, sex, age, and height were taken and filled to Emergency Nutrition Assessment (ENA) for Standardized Monitoring and Assessment of Relief and Transition (SMART) 2007 software to convert the nutritional data into Z-scores of the indices. The data was then transported to SPSS version 20. Bivariate and Multivariable binary logistic regressions were carried out to investigate the effect of each independent variable on the dependent variable. Statistical significance was set at p-value < 0.05.

**Result:**

The odds of Bitot‘s spots (1.46 %) and night blindness (1.22 %) were higher than the WHO Cut-off levels used to define a public health problem. The odds of night blindness was 4 times higher among children belonging to family size greater or equal to four [Adjusted Odds Ratio (AOR) = 4.18, 95 % CI = 1.15,15.3] and 6 times higher among children of illiterate mothers [AOR = 5.96 , 95 % CI = 1.33,26.69]. The odds of Bitot‘s spots was 5.35 times higher among children belonging to family size greater or more four [AOR =5.35; 95 % CI = 1.49, 19.2], 4.75 times higher among children of illiterate mothers [AOR = 4.75, 95 % CI =1.32, 17.18] and 6 times higher in males than females [AOR = 5.8, 95 % CI = 1.65, 20.46].

**Conclusions:**

The study revealed that night blindness and Bitot‘s spots are major nutritional problems in the study area. The independent predictors of night blindness were mother illiteracy status and large family size and also for Bitot‘s spots were mother illiteracy status, male sex of child and large family size. Therefore, the need to increase educational level of mother, use of family planning of women and emphasis on male children and children from large family size by involving the Education sector, Health sector, (Federal Ministry of Health) FMOH and (Tigray Regional Health Bureau) TRHB is crucial.

## Background

Vitamin A is a vital micronutrient involved in several biochemical activities crucial for regular biological purpose, including vision and immunocompentence. Its deficiency associated with increased death and severity of illness from respiratory and gastrointestinal disease. This is due to the abating of the immune system by the interruption of resistant barriers at epithelial and mucosal surfaces and the weakening of both humoral and cellular immunity. This effect in compromised resistance suitable to fetching infected, in addition insufficient immune response to infection. Therefore, the real extent of vitamin A deficiency is usually covered by disease in affected communities [1].

Vitamin A Deficiency (VAD) is the main nutritional concern in poor communities, particularly in low income countries. Pre-school children because of their high requirement per body weight and higher incidence of infection are the most vulnerable group to VAD [2]. Its existence as a community health problem can be evaluated by assessing the prevalence of night blindness and Bitot‘s spot in a population [3]. VAD causes night blindness and bitot‘s spot during childhood period, are indicators of increased morbidity, protein-energy malnutrition, anemia, and elevated mortality in preschool children. These were more vulnerable to illness, both infectious (e.g. symptoms of urinary infection, diarrhea and dysentery) and non-infectious (e.g. symptoms poor appetite, nausea and vomiting) [4].

Globally, around 33.3 % of preschool-age children (One third (190 million) of the world‘s preschool children) were Vitamin A Deficient and in 122 countries were a public health significance [3] and supposed to contribute to over 1 million childhood deaths a year [2]. An estimated 44.4 % of preschool children in Africa (56.4 million children) are vitamin A deficient [3].

Worldwide mainly in Africa and Asia there are 1.5 million blind children. In developing countries blindness in children is usually caused by Vitamin A Deficiency, Measles, conjunctivitis of the newborn and harmful traditional eye medicines [5]. Approximately 250,000 to 500,000 malnourished children in the developing world acquired blindness each year from a deficiency of vitamin A, nearly half of them die within twelve month of losing their eyesight [6].

In Ethiopia, Micronutrient deficiencies contribute significantly to morbidity and mortality among preschool children. Predominantly, Vitamin A deficiencies are one of the significant public health problems. In Ethiopia, vitamin A deficiency leads to 80,000 deaths in a year and affects 61 % of preschool children [7, 8].

However, research study based on information regarding night blindness, Bitot‘s spot and vitamin A intake among preschool children from the rural communities is limited. Particularly to this study area there was no previous study. Therefore, the purpose of this study was to provide information regarding the prevalence and associated factors of the night blindness and Bitot‘s spots among preschool children.

## Methods

### Study area and population

Asgede-Tsimbla is one of the administrative districts of North West Zone of Tigray regions, which are about 1107 kilometer away from Addis Ababa (The capital city of Ethiopia) and 324 km away from Mekelle (The capital city of Tigray regional state). It has 27 kebelles (Smallest Administrative Unit), seven health centers and seventeen health posts. Endabaguna is the main town of the district. In 2012/13 G.C estimated population of the district were 161,139 of which 79,270 were males and 81,869 were females. And the total under-five years of age population were 24,171 out of which 11,892 were males and 12,279 were females. The total households of the district were 36,623. The weather condition of the district is mostly “kola” (Hottest weather condition) and “Weyna dega” (weather condition between coldest and hottest) and most of the preschool children are at risk of developing infectious disease, especially malaria and pneumonia. The staple foods of the district are made mainly from “injera”; (made mainly from sorghum, teff, maize), “wot” ;( made mainly from legumes); “tela*”* ;( made mainly from millet) [9] and Data was collected from January 27 to march 7, 2014. Study population were all preschool children from eight selected rural kebelles of Asgede-Tsimbla district and who were residents of the selected kebelles of the district during the data collection period were included in the study.

### Sample size calculation

Sample size was calculated by using single population estimate formula,[10] and then the largest sample was taken for the study 3.2 % proportion of previous night blindness among children of Amhara region was considered [11] And then with the assumption of 95 % CI and 1 % Marginal errors calculated sample size was 1200 by considering 5 % contingency rate final total sample size was 1260.

### Sampling technique

Eight rural kebelles was selected randomly from the existing 27 rural kebelles. The total study populations House Hold’s (HHs) of each eight selected kebelles with preschool children were obtained. The total sample size was distributed to each of the selected kebelles households based on probability proportional to sample Size allocation. One study subject from each household was obtained and in a household with two and above children only one child was randomly selected. The study subjects (HHs) were selected by systematic random sampling until the optimal sample size was reached. The “k” value of each kebelle was calculated by dividing the total study population of each kebelle with study subjects of corresponding kebelle obtained by probability proportional to sample size.

### Data collection tool

Data was collected using anthropometric measurement and structured interview administered questionnaire & physical examination for night blindness and bitot‘s spot as a source of data. The questionnaire was adapted from different relevant studies and WHO (World Health Organization) /FAO (Food and Agriculture Organization) [3, 12] was developed in English and then was translated in to Tigrigna and back translation by different persons was done to check its consistency. Based on these data on socio-economic and demographic; maternal and child related factors; diet related factors and water and sanitation factors was collected.

Dietary diversity has been defined as the number of individual food items or food groups consumed over a given period of time and the reference period can vary, but is most often the previous day or week. Individual Dietary Diversity Score (IDDS) is a measure of the total number of different food groups eaten in the previous 24 hours by children [13]. It was collected by asking mothers did your child consume any of the following kind of foods in the last 24 hours. Did your child eat any of flat bread, sweet red pepper, cabbage, carrot, other fruits such as banana, other vegetables such as green beans, vegetable oil, beef, chicken, milk, beans, eggs and other vitamin A reach foods yesterday?

Supervisors and data collectors (clinical nurse professional) were trained on clinical assessment of vitamin A deficiency, how to perform clinical examination and data gathering regarding night blindness and bitot’s spot by principal investigators and expert ophthalmic nurse

The assessment of child night-blindness (a local term “hima” in Tigregna and “dafinit” in Amharic) was based on reports from mothers regarding the condition of their children in difficulty seeing with decreased light or at night and a standardized sequence of questions was used and for bitot’s spot children was assessed based on eye examination by trained clinical nurse examine children with collections of keratin in the conjunctiva with a small cheesy or foamy ocular lesion overlaying a patch of rough or xerotic conjunctiva usually accompanied by night blindness.

Anthropometric measurements were done by recording and measuring Age, sex, and height of children

Age: was collected from the mother and counter checked using vaccination cards, baptismal certificates or other forms of informal recording. When these recordings were not available, a calendar of locally important events was used, Sex: was recorded as female and male and Height: Height of children was measured in a standing-up position to the nearest 0.1 cm using a measuring board, the child being barefooted and free of any head wearing. Finally, by using 2007WHO growth standards Z-score system of ENA SMART software was used to calculate height-for-age (HAZ). Children Z-score below −2 were classified as stunted. It was standardized from the United States National Center for Health Statistics as the reference.

### Data quality management

It was ensured by trained data collectors & supervisors and providing day to day supervision during the whole period of data collection. Pretest was conducted in other nearby District (Tahtay koraro) and the necessary adjustments were done prior to data collection started in the actual study area. The supervisors were responsible to care of smooth process of data collection process & carry out a reliability study on the number of selected study subjects. At the end of each day, the questionnaire was checked for completeness.

### Data analysis

The data was entered, sorted; cleaned and analyzed using SPSS version 20 and anthropometric measurements were calculated using ENA SMART software (WHO growth standards Z-score system of ENA SMART software was used to calculate height-for-age (HAZ). Children below −2 were characterized as stunted) and were transferred to SPSS version 20 for analysis. Data cleaning was performed to check for consistencies and missed values and variables.

Frequency and percentage were used for summarizing categorical variables, after checking the normality of continuous variables by using histogram; mean & standard deviation were employed to present normally distributed continuous variables. Tables were used for univariate summarization of relevant variables. Bivariate logistic regression was used to see the independent effect of predictors on outcome variables.

Variable adjusted for night blindness were literacy status of mothers, family size, age of child and sex of child and for Bitot‘s spots literacy status of mothers, family size, age of child, sex of child and latrine available were also adjusted and transferred to multi variable binary logistic regression by using preset P-value of 0.25 [14].

Multi collinearity effect was assessed and the mean Variance Inflation Factor (VIF) > 10 was used as cut off point [14]. Furthermore, confounding was managed using the multivariate analysis. The final model was then tested for its goodness of fit by Hosmer and Lemeshow p-value > 0.05 was best fit. Significance was declared when p-value was < 0.05.

### Ethical consideration

Ethical clearance was obtained from the Institutional Review Board of College of Health Sciences, Mekelle University. Permission to undertake the study was obtained from Tigray health bureau to the Asgede-Tsimbla District health office and from the district to the kebelles’ administrative bodies. Caregivers or mothers of the study participant was informed about the purpose of study, anticipated benefit, how they are chosen to participate, data collection procedures and therefore full right to refuse or participate in the study and data collection procedures. And finally written informed consent was sought from individual participants before interview.

## Results

A total of 1260 mothers approached, out of this 1230 mothers were participated and only 30 mothers were not interested in this study.

### Socio-demographic and economic character tics

Table 1 shows the socio demographic characteristics of the respondents. Hence, the study participants were children aged from 24 to 59 months. Mothers’ age also ranges were from 17 to 52 years. The mean age of children and their mothers were 41.74 (SD ± 10.55) months and 30.99 (SD ± 7.7) years respectively. Majority 98.8 % were orthodox Christians in religion. Above half of the mothers 59.5 % were not able to read and write. Around three fourth of mother 75.7 % occupation was farming.

**Table 1:** Socio-economic and demographic characteristics among preschool children in rural Asgede-tsimbla District, North Ethiopia, March 2014

Variables	Frequency (n)	Percentage (%)
Age of the child		
24-35 months	404	32.8
36-47 months	406	33.0
49-59 months	410	34.1
Sex of the child		
Male	603	49
Female	627	51
Age of mothers		
less than 18	24	2.0
18-24	235	19.1
25-29	319	25.9
30-34	230	18.7
35-39	235	19.1
40-44	122	9.9
45 or more	65	5.3
Religion		
Orthodox Christian	1215	98.8
Muslim	15	1.2
Marital status		
Married	1060	86.2
Divorced	131	10.7
Single	13	1.1
Widowed	26	2.1
Maternal literacy		
illiterate	638	59.51
literate	592	40.49
Maternal occupation		
Farmer	931	75.7
Others*	296	24.3
Father literacy		
illiterate	628	51
literate	602	49
Income		
<650	586	47.6
≥650	644	52.4
Family size		
<4	620	50.4
≥4	610	49.6

*others = House wife, Private employee, Daily laborer and Merchant

### Maternal and child related variables

As shown on the Table 2 below, maternal and child related variable was presented and the corresponding percent of antenatal care follow up and post-natal care follow up were 37.8 % and 7 % respectively. 88 (7.1 %) mothers gave birth at the health institution. Nearly half of the preschool children 47 % were stunted main reason were deeply rooted in poverty and deprivation, stunting is a nutrition problem. Majority 92.4 % of the preschool children had received vaccine and 85.2 % of vitamin A supplementation.

**Table 2:** Maternal and child related variables in rural Asgede-tsimbla District, North Ethiopia, March 2014

Variables	Frequency	Percentage
Ante Natal care (ANC)		
No	465	37.8
Yes	765	62.2
Post Natal Care (PNC)		
No	86	7
Yes	1144	93
Place of delivery		
Home	1133	92.1
H. institution	97	7.9
Number of pregnancy		
≤3	754	61.3
>3	476	38.7
Number of children born alive		
<3	710	57.7
≥3	520	42.3
Start of complementary feeding		
≤6 months	998	81.1
>6 months	232	18.9
Duration of breast feeding		
6-25 months	843	68.5
26-36 months	387	31.5
Stunting status		
Stunted	578	47
Not stunted	652	53

### Water and Sanitation Variables

In this study 89.1 % of households were used protected well/spring as main sources of water. And about 74.2 %, 62.9 %, 36.3 % of households had latrine, Garbage disposal site, and hand washing basin respectively.

### Diet related factors

Milk and its products, Egg, Animal origin foods, dark green leaves, yellow vegetables and fruits should be taken three times or more per week. In this study six hundred forty three (52.7 %) children were found to be with low dietary diversity score while the remaining five hundred eighty seven (47.3 %) were found to be with high dietary diversity score.

### Prevalence of night blindness and Bitot’s spot

The number of preschool children with night blindness were 15 (1.2 %) and Bitot‘s spot were 18 (1.5 %). Out of 18 subjects with Bitot‘s spot, 7(0.6 %) subjects had night blindness while the remaining 11(0.9 %) subjects with Bitot‘s spot hadn’t night blindness.

### Associated factors of night blindness

After applying both bivariate and multivariate logistic regression, mother’s literacy status and family size were found to be independent predictors of night blindness.

As describe in the Table 3 above, the odds of preschool children from illiterate mothers were 5.9 times greater to be night blinded as compared to the preschool children from literate mothers [Adjusted Odds Ratio(AOR) = 5.9, 95 % CI = 1.33, 26.69]. Further, the odds of preschool children from ≥ 4 family size were 4 times greater to be night blinded as compared to preschool children from < 4 family size [AOR = 4.2, 95 % CI = 1.16, 15.13].

**Table 3:** Result of bivariate & multivariate (adjusted for different variables) logistic regression among preschool children in Asgede-tsimbla rural district, North Ethiopia, 2014

	Night blindness			
Variables	Yes	No	COR(95 % CI)	AOR(95 % CI)
Literacy status of mother				
illiterate	13(0.2)	625 (99.8)	6.136(1.38,27.30)	5.95(1.33, 26.69)*
literate	2(0.3)	590 (99.7)	1	1
Family size				
<4	3(0.5)	607(99.5)	1	1
≥4	12(1.9)	608(98.1)	3.99(1.12,14.22)	4.18(1.15, 15.13)*
Age of child				
24-35 months	3(0.7)	401(99.3)	1	1
36-47 months	5(1.2)	401(98.8)	1.67(0.4,7.02)	1.65(.39, 7.01)
48-59 months	7(1.7)	413(98.3)	2.27(.58,8.82)	1.69(.42, 6.68)
Sex of child				
Male	10(1.7)	593(98.3)	2.0(0.72,6.18)	2.15(.73,6.39)
Female	5(0.8)	622(99.2)	1	1

*Statistically significant (p-value < 0.05)

Unmarked = not significant

### Associated factors of Bitot’s spots

Both bivariate and multivariate logistic regression were applied to identify factors associated with bitot’s and then mother’s literacy status, family size and sex of the child were found to be independent predictors.

As describe in the Table 4 above, the odds of preschool children from illiterate mothers were 4.7 times greater to develop Bitot‘s spots as compared to the preschool children from literate mothers [AOR = 4.7, 95 % CI = 1.32, 17.19]. Further, the odds of preschool children from greater or equal to four family size were 5.3 times greater to develop Bitot‘s spots as compared to preschool children from less than four family size [AOR = 5.3, 95 % CI = 1.49, 19.19]. In addition, the odds of males preschool children were 5.8 times greater to develop Bitot‘s spot as compared to females preschool children [AOR = 5.8, 95 % CI = 1.65, 20.46].

**Table 4:** Logistic regression showing the effect of different variables on the presence or absence of current Bitot‘s spots among preschool children in Asgede-tsimbla rural district, North Ethiopia, 2014

	Bitot‘s spots			
Variables	Yes (%)	No (%)	COR(95 % CI)	AOR(95 % CI)
Literacy status of mother				
illiterate	15(2.4)	623(97.6)	4.73(1.36,16.41)	4.76(1.32,17.18)*
literate	3(0.5)	589(99.5)	1	1
Latrine availability				
Yes	10(1.1)	904(98.9)	.56(0.20,1.55)	0.43(0.17,1.09)
No	8(2.5)	308(97.5)	1	1
Family size				
<4	3(0.5)	607(99.5)	1	1
≥4	15(2.4)	605(97.6)	5.02(1.4,17.42)	5.35(1.49,19.2)*
Age of child				
24-35 months	3(0.7)	401(99.3)	1	1
36-47 months	5(1.2)	401(98.8)	1.67(0.4,7.021)	1.55(.36, 6.71)
48-59 months	10(2.4)	410(97.6)	3.26(0.89,11.93)	2.34(0.62,8.83)
Sex of child				
Male	15(2.5)	588(97.5)	5.31(1.53,18.42)	5.82(1.65,20.46)*
Female	3(0.5)	624(99.5)	1	1

*Statistically significant (p-value < 0.05)

Unmarked = not significant

## Discussion

It is vitally important to realize that many children who are vitamin A deficient will not have the eye signs (night blindness and Bitots spot). This means that children with the eye signs are only the “tip of the iceberg” there will be many other children in the community who are vitamin A deficient but who have completely normal eyes and vision [15].

In this study 1.2 % of the preschool children developed night blindness which were higher than the cut of point of public health importance of WHO (night blindness cut of point 1 %) [5]. Therefore, the difference is not statistically significant. It might be the variation in sample size. It had also similar with the study findings conducted in Madhya Pradesh (0.8 %) [16], Maharashtra (1.1 %) [17], Nigeria (1 %) [18], And some other studies conducted in Ethiopia; nationally (0.8 %) and Harari (1.1 %), Amhara (1 %), Benshangul gumuz (1 %) and Afar (0.9 %), Alaje and Samre woredas of Tigray (0.9 %) [11] this might be due to the similar study design and maternal knowledge regarding Vitamin A inadequate across the nation.

But the finding was relatively higher than the findings from Nepal (0.27 %) and Bolivia (0.2 %) [1, 19]. The possible reasons might be due to difference in the quantity of diet consumed, and it was observed that those who consumed vitamin A-containing foods daily were significantly less likely to suffer from night blindness [20].

In this study, the odds of children of those illiterate mothers were 6 times greater to develop night blindness when compared to the children of literate mothers [AOR = 5.9 (95 % CI: (1.33, 26.69)]. This could be due to the reason that inappropriate feeding practices of educated mothers may also be easily corrected to some extent during nutrition education intervention[21].

And also in the current study, children from four or greater of family size was a statistical significant at increased risk to develop night blindness when compared to the children from less than four of family size [AOR = 4.2; (95 % CI: (1.16, 15.13)]. This might be due to the reason that big family size of children had reduced their share of energy, protein, and iron and vitamin A source foods. Note that iron deficiency reduces appetite of children for foods containing vitamin A and reduced protein energy can lead to diarrhea which in turn causes night blindness as manifestation of vitamin A deficiency [8]. It is similar to the study conducted in Sudan [22]. It might be the poor intakes of animal source foods and fruits and vegetables rich in iron.

On Bitot’s spots in this study results showed that 1.5 % of preschool children were suffered from Bitot‘s spots which was around three times higher than the cut of point of public health problem importance of WHO (Bitot‘s spot cut of point 0.5 %) for considering VAD as a public health problem and signifies that it is still a public health problem among pre-school children of the study area [23] this finding was consistent with the result reported from a study conducted in Nepal (1.27) [1] and India ((Madhya Pradesh, Maharashtra and Anganwadi) (1.4 %, 1.3 %, and 1.4 %) respectively [16, 17, 24] the finding was also in line with some other studies conducted in Ethiopia; National (1.7 %) and Afar (2.1 %), Oromia (1.5 %), Addis Ababa (1.4 %), Harari (1.2 %) and Dire Dawa (1.1 %) Alaje and Samre Weredas of Tigray (1.5 %) [23, 25]. This might be due to similar study design and dietary intake might contribute to the problem of poor vitamin A status.

In this study; the odds of Bitot‘s spots among preschool children was relatively higher as compared to the result reported from the study conducted in Brazil which noted that the odds of preschool children was 0.6 % [19], the reason might be the poor intakes of animal source foods and fruits and vegetables rich in provitamin A carotenoids, coupled with a low fat intake, results in VAD, especially among young children [12] and lower as compared in Peru which noted that the odds of preschool children was 3 %. The reason might be due to some short term intervention to improve Vitamin A status of preschool children [19].

Sex of the child was found to be determinant factor for Bitot‘s spots in which male children were at increased risk when compared to the female children [AOR = 5.8; (95 % CI: (1.65, 20.46)]. And this finding was consistent with the study conducted in India [4]. There have been no convincing physiologically based studies that could account for the intrinsic factor sex difference. This suggests that cultural factors more likely explain the difference [26]. This study is consistent with the study conducted in India [4].

In the current study, children of those illiterate mothers were a statistical significant at increased risk to develop Bitot‘s spots when compared to the children of literate mothers [AOR = 4.7; (95 % CI: (1.32, 17.18)]. This might be mothers who were illiterate or undereducated often lack knowledge of the dietary needs of children. Correcting maternal literacy has been associated with improved child nutritional care [3]. This might be that literate mothers can be exposed and updated themselves to reading materials such as broachers, posters and other leaflets concerning about nutritional activities and child feeding practices; so, they can apply it to their children in order to make their children well-nourished. This was also found to be consistent with finding of a three study in India [4, 16, 24].

The association between the odds of Bitot‘s spots and family size revealed that the odds was significantly higher among the children with the family size of four or more members (AOR = 5.3 (95 % CI: (1.49, 19.2)]. This might be due to the variation in vaccination status of children that can be identified as a risk factor for clinical vitamin A deficiency [27]. A similar observation was made in India [4].

## Conclusions

The study revealed that night blindness and Bitot‘s spots are major nutritional problems in the study area. The independent predictors of night blindness were mother illiteracy status and large family size and also for Bitot‘s spots were mother illiteracy status, male sex of child and large family size.

## Abbreviations

EPI: Extended Programme of Immunization
HIV: Human Immunodeficiency Virus
IDDS: Individual Dietary Diversity Score
PI: Principal Investigator
RTI: Respiratory Tract Infection
VAD: Vitamin A Deficiency
VAS: Vitamin A Supplementation
WHO: World Health Organization
XN: Night blindness
X1A: Conjuctival Xerosis
X1B: Bitot‘s Spots
VIF: Variance inflation factor
